# Interaction between spiders and ticks—ancient arthropod predatory behavior?

**DOI:** 10.1007/s00436-024-08282-2

**Published:** 2024-07-09

**Authors:** José de la Fuente, Agustín Estrada-Peña, Marcelo B. Labruna, Matias P. J. Szabó

**Affiliations:** 1 Instituto de Investigación en Recursos Cinegéticos (IREC), Consejo Superior de Investigaciones Científicas (CSIC), SaBio, Universidad de Castilla-La Mancha (UCLM)-Junta de Comunidades de Castilla-La Mancha (JCCM), Ronda de Toledo 12, 13005 Ciudad Real, Spain; 2 Department of Veterinary Pathobiology, Center for Veterinary Health Sciences, Oklahoma State University, Stillwater, OK 74078 USA; 3 Instituto Agroalimentario de Aragón-IA2 (Universidad de Zaragoza-CITA); Retired, 50013 Zaragoza, Spain; 4 Department of Preventive Veterinary Medicine and Animal Health, Faculty of Veterinary Medicine, University of São Paulo, São Paulo, 05508-270 Brazil; 5 Laboratório de Ixodologia, Faculdade de Medicina Veterinária, Universidade Federal de Uberlândia, Av. Pará, Campus Umuarama-Bloco 6T, Uberlândia, Minas Gerais CEP 38405-302 Brazil; 6 Departamento de Patología Animal, Facultad de Veterinaria, Universidad de Zaragoza, Miguel Servet 177; Retired, 50013 Zaragoza, Spain

**Keywords:** Amber, Predatory, Spider, Tick, Zooarcheology

## Abstract

Ticks are ectoparasite vectors of pathogens affecting human and animal health worldwide. Rational integration of different control interventions including plant-derived repellents and acaricides, management of natural predators, and vaccines is required for innovative approaches to reduce the risks associated with ticks and tick-borne diseases. How tick populations are naturally controlled is always a question. Tick interactions with other arthropods including predators evolved from ancient times. In this study, Cretaceous (ca. 100 Mya) Burmese amber inclusions were identified as probably related to *Compluriscutula vetulum* (Acari: Ixodida: Ixodidae) tick larvae and spider silk. As illustrated in this study, ancient interactions between ticks and spiders may support arthropod predatory behavior as a natural control intervention. Rational integrative management of different tick control interventions including natural predators under a One Health perspective will contribute to effectively and sustainably reducing the risks associated with ticks and tick-borne diseases.

## Introduction

Ticks (Acari: Ixodidae) are blood-feeding ectoparasite vectors of pathogens affecting human and animal health worldwide (de Souza and Weaver 2024). Natural repellents and chemical acaricides are the most common tick control interventions, and recent advances in plant-derived natural compounds and anti-tick vaccines provide new environmentally sound, effective, and sustainable control interventions (Malak et al. 2024; de la Fuente and Ghosh 2024). Nevertheless, rational integration of different control interventions including management of natural predators is required for innovative approaches to reduce the risks associated with ticks and tick-borne diseases (de la Fuente et al. 2023; Machtinger et al. 2024).

Tick-host–pathogen interactions evolved with associations with other arthropods (de la Fuente et al. 2015). Spider silk in amber inclusions is a rare finding, but web fragments with prey and silk strands with glue droplets have been reported (e.g. Zschokke 2003, 2004; Peñalver et al. 2006; Boucot and Poinar 2010; Ross and Sheridan 2013; Dunlop et al. 2018). Putative predated arthropods by spiders found in amber inclusions included myriapods, pseudoscorpions, insects, midges, mites, and ticks. Tick fossils are also rare findings in amber (e.g., de la Fuente 2003; Mans et al. 2016; Peñalver et al. 2018; Dunlop et al. 2018), and only one report has provided evidence associating ticks with predatory spiders found in Cretaceous amber (Dunlop et al. 2018).

In a Corsican house invaded by both kennel ticks *Rhipicephalus sanguineus* and Theridiidae spiders *Teutona triangulosa* (Walckenaer 1802), the spiders were observed feeding on ticks (Sautet 1936). Under experimental conditions, they fed on both the immature and adult stages, and young spiders attacked ticks shortly after hatching. Taken together, this evidence is supported by current reports of arthropods including spiders as predators of ticks (Samish and Alekseev 2001; Bernardi et al. 2010; Fischhoff et al. 2018).

To provide further information on the ancient interactions between ticks and spiders, herein we analyzed inclusions in Burmese amber.

## Materials and methods

### Amber inclusions

**Fig. 1:**
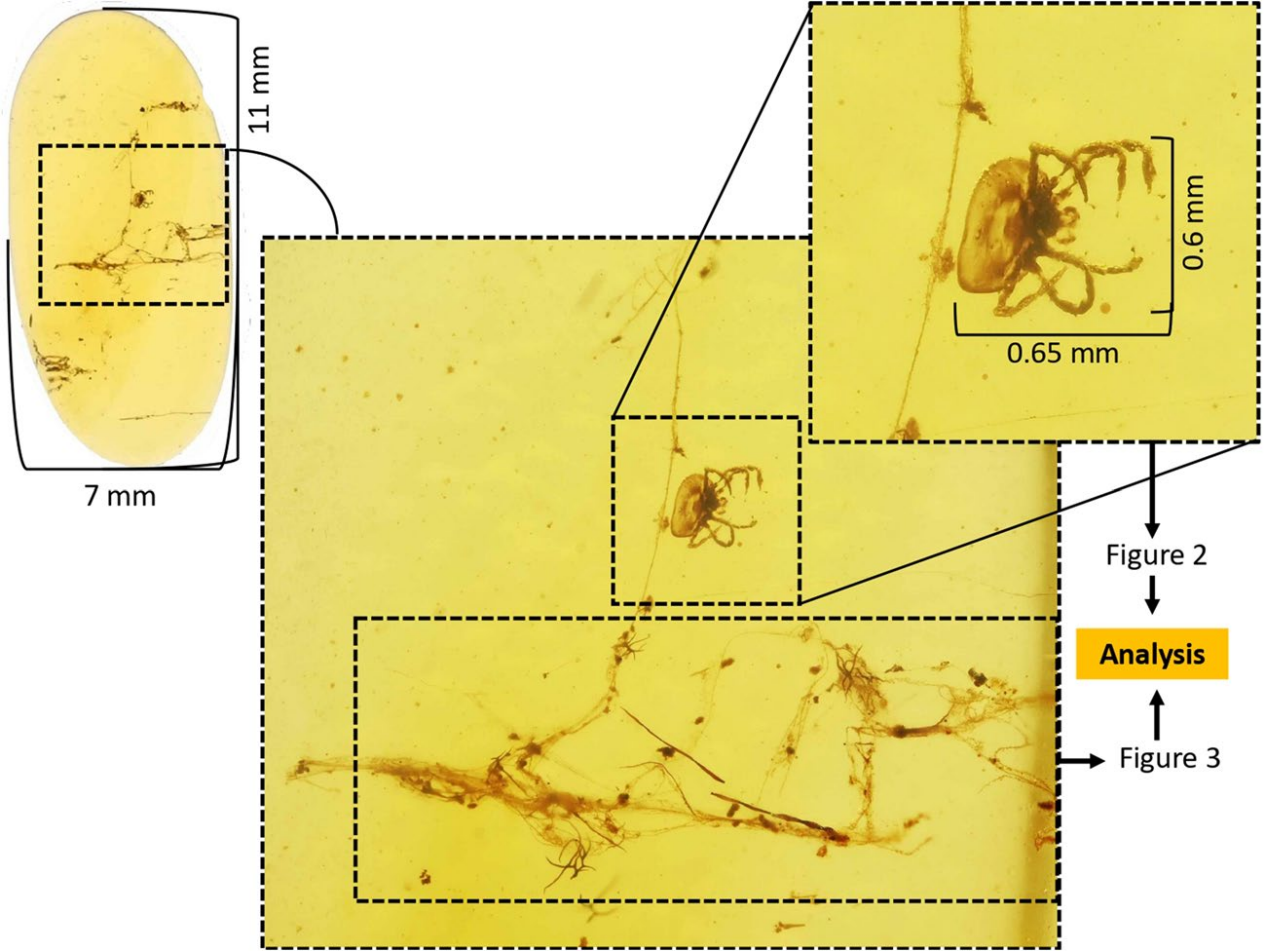
Amber inclusions. Cretaceous Burmese amber (ca. 100 Mya) with arachnid and spider silk strands. Amber piece is shown and the main inclusions in the rectangle are highlighted with the parts used for analysis in Figs. 2 and 3

Inclusions in Burmese (Burma, Myanmar) amber (Cretaceous, ca. 100 Mya) with Arachnida (Lamarck 1801) and spider (Araneae) silk strands were used for the study. The amber piece originated from the KGJ Collection (Ciudad Real, Spain) (Fig. 1) and was dated to the late Cretaceous by radiometric analysis (99.13 ± 0.82 Mya; Shi et al. 2012).

### Image capture and analysis

Images were captured with a Leica (L’Hospitalet de Llobregat, Barcelona, Spain) M80 routine stereo microscope using a 1X PLAN objective and a 2–6 × zoom (https://www.leica-microsystems.com/products/light-microscopes/stereo-microscopes/p/leica-m80/), a Carl Zeiss stereomicroscope (SteREO Discovery V12, Munich, Germany) using the ZEN 2 pro software. Microscope images were analyzed using the ImageJ program (https://imagej.net/ij/) and pencil sketches of images using IOimageonline.co (https://pencilsketch.imageonline.co/index.php).

## Results and discussion

**Fig. 2:**
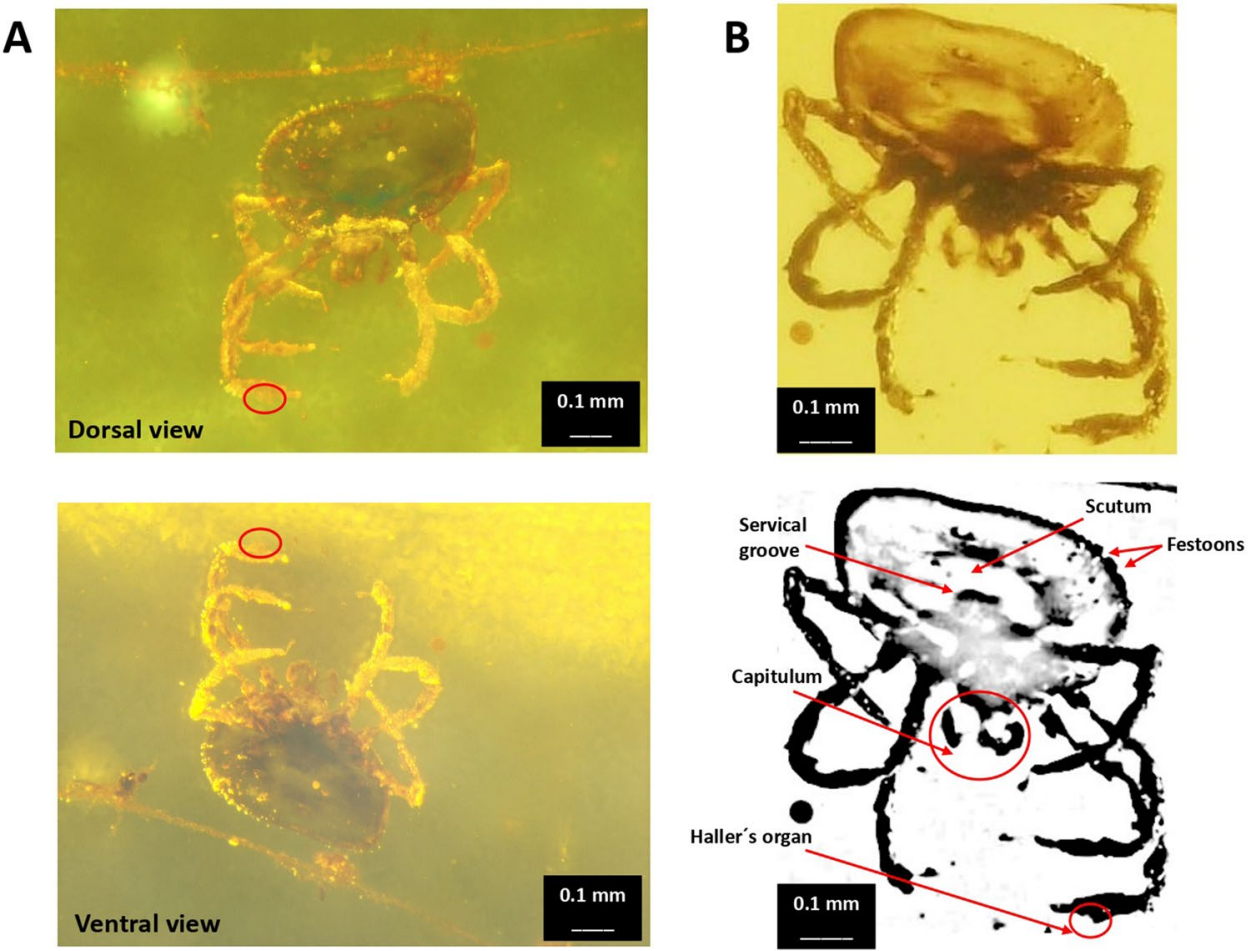
Arachnid amber inclusion. **A** Dorsal and ventral views. **B** Interpretative camera drawings. The red circle delimitates the Haller’s organ, visible as a typical protuberance on the dorsal surface of Tarsus I

**Fig. 3:**
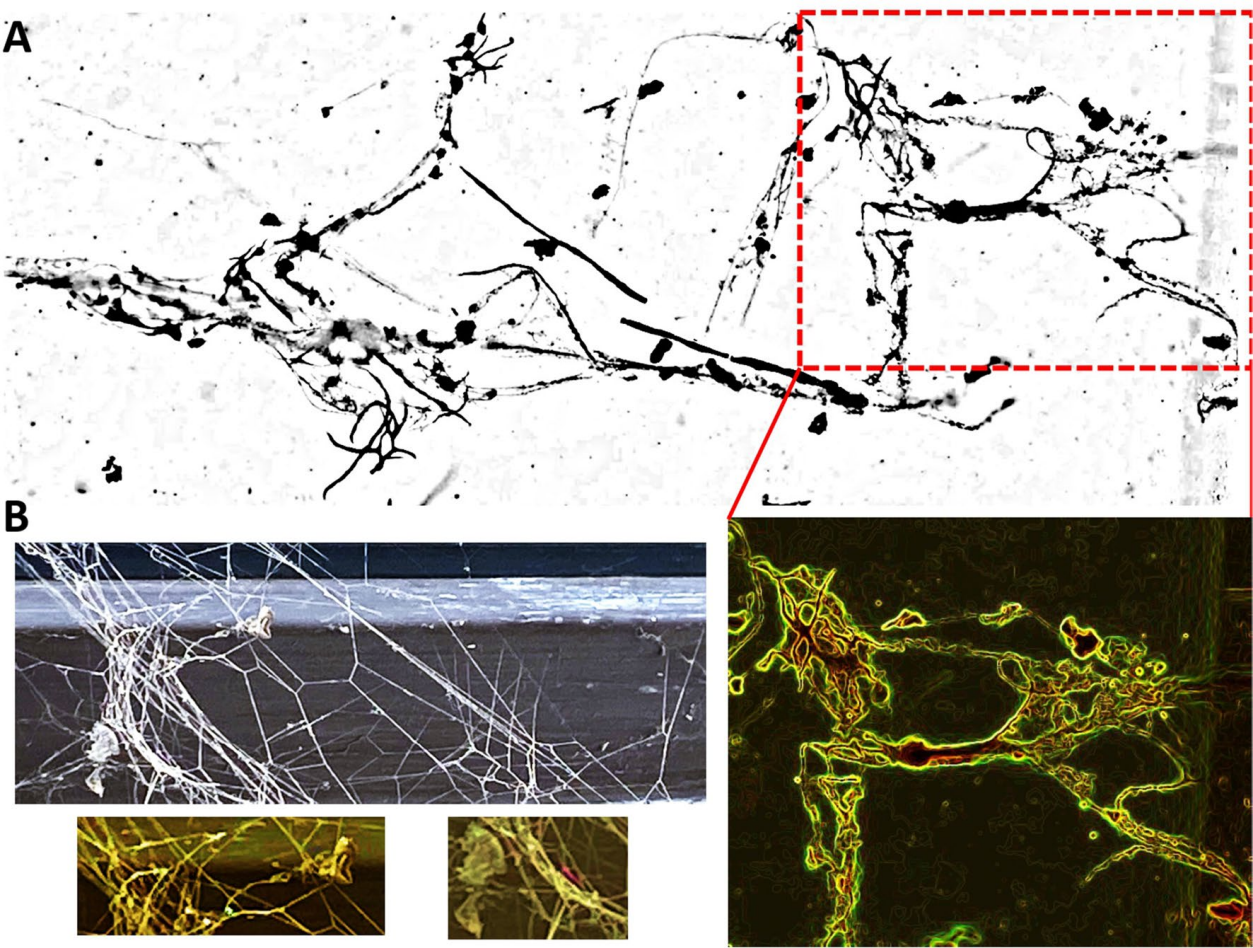
Spider silk strands. **A** Interpretative camera drawings of the spider web inclusion. The red rectangle represents part of the ancient spider web shown also in color below the rectangle. **B** Modern comparisons with spider web from inhouse in Shanghai, China, collected March 21, 2024

The results initially suggested that arachnid inclusion may correspond to tick (Ixodidae) larvae or mite (Holothyrida) (Fig. 1). Then, the analysis of arachnid dorsal and ventral views (Fig. 2A) and interpretative camera drawings (Fig. 2B) supported evidence of the tick larvae. Diagnostic was based on a circular body, absence of eyes and anal groove, festoons on the body’s left side dorsal view, segmented palpi in the capitulum, and presence of Haller’s organs (Fig. 2A and B). Based on previous findings in Burmese amber (Poinar and Buckley 2008), the inclusion may be related to *Compluriscutula vetulum* (Acari: Ixodida: Ixodidae). However, we cannot confirm it with certainty due to the impossibility of verifying key characters related to *C. vetulum* such as the presence of 13 festoons and the 2/2 hypostome dentition (Poinar and Buckley 2008). Based on the silk strand structure (Fig. 3A and B), fungal hyphae were discarded.

Although the results do not allow the identification of the spider family and do not demonstrate spider predation of a tick, evidence suggests ancient interactions between spiders and ticks with possible predatory behavior. Considering data from Dunlop et al. (2018), this study and future findings may allow the establishment of a coevolutionary relationship between spiders and ticks with a possible role of arthropods in the natural control of tick populations.

Learning from natural tick predators and their evolutionary relations may suggest new rational measures for tick control. Integrative management of different tick control interventions including natural predators and vaccines will contribute to effectively and sustainably reducing the risks associated with ticks and tick-borne diseases. However, the possible impact of climate change on tick and insect abundance and biomass composition should be considered (Müller et al. 2024; van Klink et al. 2024).

## Data Availability

No datasets were generated or analyzed during the current study.
